# The ethnicity attainment gap among medical and biomedical science students: a qualitative study

**DOI:** 10.1186/s12909-018-1426-5

**Published:** 2018-12-29

**Authors:** Hugh Claridge, Khadija Stone, Michael Ussher

**Affiliations:** 1 Population Health Research Institute, St George’s, University of London, Cranmer Terrace, London, SW17 0RE UK; 2 Swansea University Medical School, Grove Building, Singleton Park, Sketty, Swansea SA2 8PP UK; 3 Institute for Social Marketing, University of Stirling, Stirling, FK8 1NZ UK

**Keywords:** Attainment gap, Black, Asian and minority ethnic, Ethnicity, Medical student, Biomedical sciences student, University, Undergraduate, Qualitative research

## Abstract

**Background:**

Black, Asian and Minority Ethnic (BAME) medical students and professionals frequently underachieve when compared with their White counterparts not only in the United Kingdom, but across the globe. There is no consensus for the definitive causes of this attainment gap, but suggestions contributing towards it include: increased feelings of isolation as a member of a minority culture or religion; a poorer higher education (HE) experience compared with White counterparts; and stereotype threat, whereby students underperform in exams from the stresses of fearing confirming to a negative-stereotype.

**Methods:**

The aim of this study was to gather qualitative data on HE experiences of medical and biomedical science students to explore factors contributing to the attainment gap. Audio-recorded, semi-structured interviews and a novel approach for this research area of ethnically-homogenous student-led focus groups, were held with students and staff at a healthcare-based university in London, where lower attainment, slower rates of degree completion and lower levels of satisfaction with HE experience were identified in BAME students compared with White students. Thematic analysis was used to manage, summarize and analyse the data.

**Results:**

Forty-one students and eight staff members were interviewed or took part in focus groups. The student data were best explained by two main themes: social factors and stereotyping, whilst staff data were also best explained by two main themes: social factors and student and staff behaviour. Social factors suggested ethnically-defined social networks and the informal transfer of knowledge impacted academic performance, isolating minority groups from useful academic information. BAME students may also be at a further disadvantage, being unable to attend social and academic functions for cultural or family reasons. Black students also mentioned changing their behaviour to combat negative stereotypes in a variety of contexts.

**Conclusions:**

This study suggests that forms of discrimination, whether conscious or unconscious, may be negatively impacting the abilities of BAME students both in examinations and in coursework choice. It highlights the importance of social networks for the transfer of academic knowledge and the impact ethnicity may have on their formation, with issues around segregation and the sharing of information outside defined groups.

**Electronic supplementary material:**

The online version of this article (10.1186/s12909-018-1426-5) contains supplementary material, which is available to authorized users.

## Background

Black, Asian and Minority Ethnic (BAME) medical students and professionals frequently underachieve when compared with their White counterparts over their education and career trajectories not only in the United Kingdom (UK) [1, 2], but across the globe [3, 4], even when other demographic variables have been adjusted-for [5]. Indeed, similar disparities in achievement can also be found for many other courses [6–9].

There is no consensus as to the definitive causes of the BAME attainment gap, but some suggestions as to what may contribute to it include: increased feelings of isolation due to being a member of a minority culture or religion [9, 10]; being less satisfied with their higher education (HE) experience than their White counterparts [11]; stereotype threat, where those from negatively-stereotyped groups can feel so anxious at the possibility of conforming to a stereotype that they underachieve in examinations [12, 13]. There is consensus that direct discrimination and examiner bias are unlikely to be sole causes, as anonymously-marked multiple choice examination results have shown similar disparities between White and BAME students [1, 14]. Thus research into this area increasingly focusses on understanding the students’ experiences and opportunities [2].

We sought further contemporary data at a healthcare-based university in London (hereafter referred to as ‘the University’), where consistent with other institutions in the medical HE sector (e.g. [15]), lower attainment, slower rates of degree completion and lower levels of satisfaction with their HE experience have been found for BAME students compared with White students. The design of the study replicates previous peer-reviewed research into this issue and also extends this research with a novel approach, by including ethnically-homogenous student-led student focus groups.

## Methods

### Aim

The aim of the study was to gather qualitative data, via audio-recorded, semi-structured interviews and focus groups, with students and staff at one London, UK healthcare-based university in order to explore their perceptions of factors contributing to the attainment gap found between White and BAME students.

### Design

A qualitative descriptive methodology was chosen, allowing an exploratory, in-depth and non-hypothesis-driven approach to eliciting a rich description of experiences and events relating to individual students and staff. A combination of focus groups and one-to-one interviews was chosen to allow student participants to choose whichever they preferred, due the potentially sensitive nature of discussing personal experiences. Focus groups were led by student members of the research team and were ethnically-homogenous (albeit in terms of broad ethnic categories) on account of findings that individuals appear to feel more at ease and are more likely to share controversial views relating to ethnic differences in groups of homogenous ethnicity than when with a mix of ethnicities [16]. Exclusively one-to-one interviews were chosen for staff due to opinions expressed at a research planning meeting with staff, who felt they would be more open when alone than with colleagues.

### Participants

An invitation to participate in the study was emailed to all ‘home’ students (ordinarily resident in the UK and with British citizenship) on undergraduate biomedical sciences and medicine courses (*n* = 1862) at the University, with some recruited through direct contact with a member of the research team, student union representatives, and members of University societies who advertised the research. Overseas, non-home domiciled and other healthcare course students were excluded due to the potential for identification given the small number of such students. Participants for six focus groups were purposively recruited by Asian/Asian British (‘Asian’), Black/African/Caribbean/Black British (‘Black’) or White: English/Welsh/Scottish/Northern Irish/British (‘White’) ethnicities, with one-to-one interviews offered as alternative to a focus group. Academic and administrative staff were emailed on an individual basis having been selected due to having regular, direct contact with students. Among the academics, these included a range of roles, from lecturers to deans. The invitation provided contextual information for why they were being contacted and explained that the research was being conducted to try and find out potential causes of the gap in student attainment between students of different ethnicities and that their participation was entirely voluntary and any information shared would be kept confidential. Students were offered an incentive for taking part of either 10 points towards an institutional academic award scheme or a £10 gift voucher. Those expressing interest were sent a participant information sheet explaining the purpose of the research, their freedom to withdraw at any time and the confidential nature of the interviews and focus groups. Those agreeing to participate were asked to complete a written consent form at the focus group or interview and a voluntary demographic information sheet.

### Interview topic guide and procedure

Topic guides for all semi-structured student focus groups and interviews and semi-structured staff interviews included a combination of open-ended and closed questions developed by the researchers through consultation with students and senior academic and widening participation staff, as well as through referring to literature on the BAME student attainment gap (e.g. [15]). These were not pilot tested. The theory of stereotype threat [12, 13] informed the content and wording of several questions. The student topics included: influences to study their chosen course; how they found integrating at the University; how they found interacting with students and staff; whether they thought their course’s content was appropriate; and whether they were aware of any discrimination or prejudice against themselves, other students or staff. The staff topics covered: awareness of any discrimination towards students or staff; personal biases or prejudices that may impact interactions with students; and awareness of any ethnic differences in learning practice and academic performance (see Additional file 1 for the full Student and Staff Interview Topic Guides).

One researcher conducted the student and staff interviews, which were all one-to-one, and was present for all the student-led focus groups (HC, male, 27 years-old, White ethnicity, master’s degree, researcher in public health, with experience of interviewing and focus groups), who was not known to the students prior to their interview invitations and had no vested interest in the research topic. HC introduced himself as a University researcher and participants were asked not to repeat anything discussed outside of the group. The focus groups were led by SL (female, Black African, 20 years-old, 2nd year biomedical sciences student, trained in leading focus groups, known to the participants) and KS (female, Asian other, 21 years-old, 3rd year biomedical sciences student, trained in leading focus groups, known to the participants). The interviews and focus groups took place in a private room in the University, were audio-recorded and transcribed verbatim by an external transcriber and subsequently anonymised. HC also made field notes during the focus groups and interviews. Transcripts were not returned to student participants for comment or correction, however some staff participants requested having advance sight of the context of their quotes if they were used. Interviews for staff and students were continued until data saturation was reached, which we were able to ascertain as thematic analysis was ongoing throughout the study, thus enabling us to note that similar points and issues were being raised with no new themes identified in the final few interviews. Ethical approval was granted by the University’s Research Ethics Committee. The Consolidated Criteria for Reporting Qualitative Research (COREQ) tool was used to ensure comprehensive reporting of the methods and findings [17] (see Additional file 2).

### Analysis

Thematic analysis was used to manage, summarize and analyse the data [18]. This enabled the researchers to gain insight into the views and experiences of each participant, while also identifying differences between participants. Thematic analysis was ongoing during the study [18]. Initial coding was undertaken independently by two researchers (HC, MU), who read and familiarised themselves with the transcripts and assigned initial codes and categories, similar codes were grouped and combined to create themes. Themes were reviewed, refined and labelled through discussions (HC, MU) to ensure that they accurately reflected the data. A hybrid of both inductive and deductive approaches was used, as the topic guide influenced the data collection and subsequent analysis, whilst the open-ended nature of the questions enabled the participants to share experiences beyond the areas covered in the topic guide. Software was not used to aid the analysis.

## Results

A total of 39 biomedical science students and 44 medical students expressed an interest in taking part, with 64 subsequently arranging an interview or focus group. However, for 23 of these, either no suitable interview time could be arranged or no further responses were received, resulting in a total of 41 student participants with mean (SD, range) age = 21 (2.78, 18 to 31). Interviews were conducted with 24 students (12 female, 12 male, nine biomedical sciences, 15 medicine), with the following from each ethnicity: Asian: Bangladeshi (1), Chinese (1), Indian (5) Pakistani (3) and Other (2); Black: African (2) and Caribbean (1); White: (8) and Irish (1) and recordings lasted for a mean (SD; range) of 33:06 min (11:25; 11:41 to 51:32). Two focus groups (one of six female biomedical science students of Black ethnicity, and one of five female medical students of Black ethnicity) were led by SL and one focus group (six biomedical science students of Asian/Asian British ethnicity, one female and five males) was led by KS. SL and KS were known to their respective focus group participants. The three remaining focus groups (for Asian medical students and White biomedical science and medical students) were abandoned due to insufficient participants. Some anecdotal evidence suggests that some students did not wish to take part in the research because of concerns about confidentiality, despite being reassured of the anonymity of their contributions. Focus groups lasted for a mean (SD; range) of 71:42 min (20:08; 49:44 to 89:48). Eight staff were interviewed (six female, two male, five academic, three administrative) with the following from each ethnicity: Asian: Chinese (1), Pakistani (1); White British (3) and the remainder did not wish to specify. Staff interviews lasted for a mean (SD; range) of 29:05 min (09:32; 17:10 to 44:00).

**Fig. 1:**
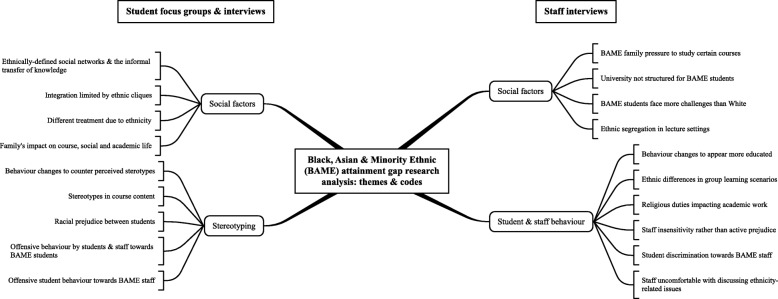
Mind map of themes and codes

Following discussion between HC and MU, it was agreed that the student data were best explained by two main themes, which are described below: (i) social factors and (ii) stereotyping. Staff data were best explained by (i) social factors and (ii) student and staff behaviour (see Fig. 1). It is clear that the topic guide structured the data collection, meaning some of the identified themes were fairly closely related to the areas covered in the topic guide. However, the open-ended nature of the questions enabled the participants to share experiences beyond the areas covered in the topic guide, allowing them to bring up topics of particular importance to themselves. To maintain participant anonymity, individual student age and precise staff University positions cannot be provided. The quotes are tagged with information on the participant who provided it, with the first character identifying whether it was in a focus group (F) or interview (I) setting; the second, third and fourth characters showing the course of the student or if staff whether they were academic or administrative (Med for medicine, Bio for biomedical sciences, Aca for academic staff, Adm for administrative staff); the fifth character in superscript shows whether female (^f^) or male (^m^); and the sixth character in superscript applies only to students and identifies whether the student self-identified as of an Asian (^a^), Black (^b^) or White (^w^) ethnicity. Therefore, the tag ‘IBio^fw^’ is a quote from an interview with a biomedical sciences student who is female and White, and the tag ‘IAdm^m^’ shows an interview with an administrative member of staff who is male. We have not indicated more specific ethnicity subgroups as no marked differences were noted between these subgroups.

## Results for student interviews and focus groups

### Social factors

Data relating to social factors covered two main issues: social networks and the informal transfer of knowledge, and the influence of family.

Student networks appeared to have both social and academic impacts, with social integration limited either by individuals being actively excluded by members of a group or through individuals assuming they cannot be part of the group due to perceived differences. Academic knowledge appears to be informally transferred between students based around, often, ethnically defined social networks. Students outside of these networks can feel excluded and actively biased against:

*FBio* ^*fb*^ *: …obviously in every uni certain groups have more access to certain things than others… [religious student society] also have a lot of access to a lot of things and for me that’s fine, if everyone is willing to share, and people are just not!*

*FBio* ^*fb*^ *: Yeah, and I think in terms of academics, they’re [religious student society] such a tight knit community, they share all their resources together…we’ve only got the little trickles that have come out, so imagine how much they’ve got within that community.*

Many students mentioned that, whilst the University is very diverse, they felt integration was difficult due to apparent ethnic divisions, and this was seen in both social and academic settings:

*IBio* ^*fb*^ *: I found people weren’t so open to, like, let’s say, make friends outside of their cultural groups [background and religion]. Yeah, and that’s just how it’s been ever since… I just found it was quite cliquey in a sense with their cultural groups*

*IBio* ^*fa*^ *: there is definitely: like, the White people sit in one corner, the Black people will sit in another corner and then the Brown people just sort of disperse between it.*

Some students also felt that they were treated differently by other students because of their ethnicity, but couldn’t be certain of it:

*IMed* ^*ma*^ *: …sometimes you just noticed certain kinds of groups of people are not really interested in that [having lunch with you], or treat you a certain way, or might be a little bit more cold or harsh, or not even kind of acknowledge you, kind of thing… sometimes it is quite hard not to wonder whether it is due to something as simple as skin colour or race, or even the way you speak or whatever.*

Due to their ethnicity, some students felt uncomfortable about attending events, such as lectures and revision groups, organised by student societies, and widely regarded as considerably better than those offered by the University:

*IBio* ^*mw*^ *: It is my personal belief of a kind of education being secular to an extent, because the [student society] does inherently great things and stuff with the power that they have, but it is still…quite closed off. I wouldn’t dream of going to a social [student society] event so much. Or I might but I would go in there knowing that I’d be the only White person there!*

Family was mentioned as a motivator for choosing medically-related degrees for a variety of reasons, including having parents in medically-related fields, or due to exposure to medicine through family illness:

*IMed* ^*ma*^ *: I think my parents really influenced me a lot. They’ve probably influenced me since I was a kid; it’s just something like bringing in topics, “Oh, so what do you want to be when you’re older? You should be a doctor!”*

*IMed* ^*mw*^ *: …my uncle…similarly my best friend was treated for some quite serious things, when I was choosing what I wanted to do with my life, and so it seemed that a lot of people who had helped me, and my friends and family in my life, were part of the medical profession and that’s seemed like something I wanted to do as well.*

However, not all family influences were seen as positive. Family responsibilities were thought to impact the amount of time students are able to attend the University, due to caring, chores or curfews, and this seemed to impact both their social and academic lives:

*IBio* ^*fa*^ *: Well, I was a commuter, so I had to work extra hard to try and make my group of friends…and also I had curfew because Asian parent problems…by the time I get home, I’m just really sleepy and then I don’t have time to, or the effort to just continue with my work.*

Some Asian students mentioned feeling that family responsibilities are not the same as for other ethnicities, particularly White students, but they’re seen as normal for Asian students:

*FBio* ^*ma*^ *: During term time, I went home as often as I could...I guess I have responsibilities that they don’t see that I have at home…*

*FBio* ^*fa*^ *: And you don’t realise how much responsibility you have because it’s just normal. So like looking after my sister, I do the shopping, cleaning the house, sorting my mum out when she needs it, things like that are really normal…*

*FBio* ^*ma*^ *: Any other person, if that was a White parent for example, they would have demanded something, but for me it’s just like actually having to do this.*

### Stereotyping

This included experiencing or witnessing stereotyping and prejudice by other students, by University staff and on clinical placements, as well as issues with course content and includes perceived or anticipated stereotyping.

Behaving ‘professionally’ when on clinical placement or during assessments with patient-actors was only raised by Black students, perhaps using it as a means of counteracting anticipated negative stereotypes:

*IMed* ^*mb*^ *: …the best way for me to deal with it [racial prejudice] is just let’s keep it business, let’s keep it professional. You know, what do we need to do today? OK, what do you need help with in that setting?*

Changing their behaviour to combat stereotypes was highlighted repeatedly by Black students in a variety of contexts, such as having to work extra hard just to be seen to be equally as capable as their non-Black peers. This is may lead to increased stress and unrealistic workloads:

*IMed* ^*mb*^ *: ...I always feel that anyway generally in life and OSCEs [Objective Structured Clinical Examinations] and assessments that I’m very conscious that I’m Black, and I’m very conscious that I have more to prove…Because I’m Black…I have to work two, three, four, five, ten times as hard to make sure that I’m seen on the level [as others]*

*FMed* ^*fb*^ *: …you have to be exceptional to be considered average!*

*FMed* ^*fb*^ *: …you just have to fight back much harder [than White male students] to get the learning that you need.*

*FMed* ^*fb*^ *: I have learnt to try and almost manage myself and manage it so that people aren’t offended by who I am. And I think you have to do that a lot more in medicine….as a Black woman, than you do as any other!*

This behavioural change extended to some Black students adopting the behaviour of White male students:

*FMed* ^*fb*^ *: …if you consider yourself to be Tom and Luke, if you get knocked back, you get up because that’s the stereotype, like a middle class White boy will get things wrong a million times, but you can’t tell him he’s got it wrong, because he’ll just give you another answer!*

Consciously being quieter than usual was raised by Black female students as a means of combatting the perceived ‘angry Black woman’ stereotype. They also mentioned being afraid to complain because of that stereotype:

*FBio* ^*fb*^ *: That angry black woman stereotype (murmurs of agreement) it really, really annoys me because it’s like sometimes I have a legitimate reason to be upset or angry with what you’re saying and it’s like, oh, there she goes again! And it’s so dismissive of the feelings and it’s like, what’s the point then? (murmurs of agreement)*

*FBio* ^*fb*^ *: I think that’s when it’s hard to speak up because I don’t think anyone ever expects a Black woman to feel bullied, they would see her as the bully.*

*IMed* ^*fb*^ *: …just because I’m from South London and I’m Black, it doesn’t mean I’m going to be really rude or stand-offish. And I have to be very open and very nice and polite, and avoid conflict with staff and with peers.*

These students also mentioned trying to dress smartly to dispel perceived scruffy stereotypes and feeling they have to put more effort in to their hair and general appearance than other ethnicities:

*IBio* ^*fb*^ *: …there may be stereotypes linked to Black people that…we’re just a bit scruffy or not! So I just know for myself, I like to make sure that I combat that stereotype by looking presentable…*

It was also clear that some students who perceived being negatively stereotyped also stereotyped other students:

*FBio* ^*fb*^ *: Iranian students are actually very much a certain type of way as well, and they shout over…they’re very rich, fair skinned so they can just go through this world not really thinking about their actions and the wider world.*

Some students had also been made aware by members of staff that they were potentially being marked-down on account of examiners’ lack of awareness about different ethnicities’ reaction to stressful situations, such as it being harder for some to detect blushing in non-White students:

*FMed* ^*fb*^ *: I get really anxious during OSCEs and my personal tutor said to me, “I remember when you are anxious, you look blank to a lot of people.” So because I’m not bright red and presenting anxiety...in the way they’re used to seeing anxiety, they don’t see me as anxious… they just see you as cool or like cut off or distance. And that affects the mark…we’re never going to be able to walk into an OSCE and look bright red; it’s just not going to happen.*

There were several instances where racially prejudicial behaviour was noted as being between students, including:

*IBio* ^*fa*^ *: …one of the girls in my year who wears the hijab but is clearly more practising than I am, made a comment about me not wearing the hijab, therefore I’m not a real Muslim, and therefore anything I was saying about Islam is just not relevant and not accepted…when you’re a Muslim and you don’t wear the hijab, or you do certain things that aren’t Islamic, you get put into a category by fellow Muslims, and then that can lead to stereotyping in a certain way, like that kind of coconut example, like, “Oh, she’s a coconut!” that kind of thing.*

Offensive behaviour by some students towards BAME staff was recounted by one focus group:

*FBio* ^*fb*^ *: The way that other students treat [two Black lecturers], they’re so disrespectful…and they make fun of their accents.*

*FBio* ^*fb*^ *: And then when [Black staff member] left they started making fun of her, being like, “Oh, she’s so angry!” and they made fun of her accent again.*

Some offensive behaviour was noted as coming from staff to students:

*IMed* ^*fa*^ *: …with [Pakistani student] he went, “What’s your real name?” and [Pakistani student] was like, “My name is [name]” And he was like, “No, no, no, what’s your real name?” because of his skin colour.*

*IBio* ^*fa*^ *: …he did complain a lot about the Asian kids [at child’s school] doing certain subjects as opposed to others, because of parental influence and stuff, and then he complained about how he thinks [area] is now full of Asian people…*

Course content was also raised as a way in which stereotypes were observed, such as through the language used when describing certain ethnic groups and the examples used for ‘typical’ cases for certain, often negatively-perceived, medical conditions:

*IMed* ^*mb*^ *: Well, let’s put it this way, when you’re in an exam and you see a mental health patient who is Caribbean, there’s only one diagnosis you’re thinking of…you know you pick schizophrenia every time. Or you know as soon as you’re reading the vignette, you know it’s going to have Caribbean, Black, weed/cannabis…I know there are a lot of people who come to uni who are not necessarily from diverse places… they’ve not…interacted within that diversity. So that doesn’t do anything to challenge prejudices or stereotypes, it only supports it.*

## Results for staff interviews

### Social factors

Family was often raised as having a potentially negative academic impact on students, encouraging students to study courses they otherwise would not have chosen. This was especially thought to be the case for families of Asian ethnicity:

*IAca* ^*f*^ *: Particularly those from Indian, Pakistani and maybe Bangladeshi backgrounds where either because the family are from a medical background…so I think it’s a combination but they seem to feel a bit pressured.*

However, staff emphasised that family pressures could also be found in other groups, and were uncomfortable with assigning specific issues to specific ethnicities:

*IAdm* ^*m*^ *: But then I can think that there’s also White students who say that [i.e. family pressure to study medicine], so every time you generalise, you think of an exception, where the family have the same sorts of prejudices.*

Some staff were aware that students have many family circumstances that could potentially have a negative academic impact, and some of these could be related to ethnicity:

*IAca* ^*f*^ *: There are other factors, such as if your parents don’t speak English and then your parents get ill, and then you need to go and translate at hospital appointments and things like that, that might be pulling people away from the course as well.*

However, this member of staff felt that family responsibilities should not necessarily receive special dispensation from the University due to the types of jobs the students are being trained for:

*IAca* ^*f*^ *: I think there is a need from the university to kind of set boundaries really because it’s about modelling professional practice in future. And trying to facilitate students making choices about to what extent they should be spending their time supporting the family and to what extent they should be on the course…So I think there is some tension between being endlessly flexible and producing safe healthcare practitioners.*

Some staff also felt that the University isn’t really structured around students from a diverse range of backgrounds and the different demands these differences place on students:

*IAdm* ^*m*^ *: I think we make assumptions based on the fact that we’ve got White 22 year olds in training to be medics, as opposed to a whole bunch of different people from a whole different range of backgrounds*

This could result in those from non-White backgrounds having fewer opportunities to socialise with both students and staff:

*IAdm* ^*m*^ *: I think it’s particularly difficult for students who don’t drink in terms of social opportunity via the SU [Student’s Union], who are very keen to have a scholarly post-work pint in the bar or one of any number of places nearby, which really does exclude a whole chunk of students from coming out… I think when you’ve got very senior academic staff who have spiders on their tie, or cobras on their tie [drinking society ties]…I think it probably sends a message…that there is something fundamentally [University] about being a member of Cobras, and there’s something fundamentally other-ing about not.*

Further challenges facing non-White students and their social and academic support networks was raised, with privately-educated White students seen as the most able to join a variety of social groups, with other students at a distinct disadvantage:

*IAca* ^*f*^ *: So, there’s something about if you’ve been at a White grammar school or a White public private school, then you know how to join ‘the club’ and what to do, and that there doesn’t seem to be that same support network necessarily for Black and Asian students. Although I would say that the Muslim student network ISOC [Islamic Society] is now emulating that very well…I think that Black students are much more isolated…because what they talk to me about is the fact that they don’t feel as comfortable because their experiences of their networks have often been church, and their White counterparts are kind of slightly dismissive of religion and church. So they’re not sure then where they fit in, so it’s a matter of kind of trying to find their support network.*

This ethnic separation of students was also noted in lecture settings, but in the form of self-segregating by ethnicity, although it was also sometimes by course too:

*IAca* ^*f*^ *: …You can see the friends groups are maybe perhaps different…Well, it’s broken down by ethnicity but then also I do lectures that are mixed, sort of inter-professional in the sense of different degree courses, and you can sometimes see them slightly, occasionally segregated [by course]*

However, another member of staff mentioned that in discussions with other staff, student segregation was more down to which students were immediately and more obviously identifiable, rather than necessarily by ethnicity:

*IAca* ^*f*^ *: …when you talked more to them [staff] about who those cliques were, the cliques were women wearing hijabs. So the cliques are the ‘other’ that we can see, so therefore there weren’t White students being talked about as cliques, but people often who all play rugby will all sit together weren’t being described in that same pejorative way.*

The University’s student population was frequently described as very diverse, but this was not seen as the case for the staff population, leading to a feeling that perhaps students from certain ethnicities do not have enough role models in senior positions at the university:

*IAca* ^*f*^ *: I would say it’s a particular issue for people of Black Caribbean heritage, because traditionally they’ve come from lower socioeconomic classes, so there’s intersectionality around class and ethnicity as well, which means that it’s very difficult for them to have existent role models, and it would be really nice to have more of those in university.*

### Student and staff behaviour

Student ethnicity was seen as impacting behaviour in a variety of ways in different academic settings. A member of staff described how some BAME students change how they speak during evaluations in order to try and seem more ‘like a doctor’ in the eyes of the examiner, but this negatively impacts them as it causes communication issues:

*IAca* ^*f*^ *: And in working with Black students too, what I’ve noticed is that their perception of how to be a good student is to use complex English language that will make you sound more important and make you sound more like a doctor should, and then that trips them up.*

Other ethnic differences in behaviour were noted in small group work, such as certain ethnic groups being quieter than others; however, it was emphasised that it was not limited to just this group:

*IAca* ^*f*^ *: I have noticed a couple of cases where particularly Oriental students…tended to have a slightly different character. They sometimes struggle with some of the group work, but that might just be their personality though, they could be shy. So there are some elements of cultural difference that I see, but otherwise, it’s just individuals I think.*

Religious duties such as set prayer times, often associated with BAME students, were reported as causing academic issues for one student, with the potential for this same issue to be impacting many more; however, it poses a challenge for the University:

*IAca* ^*f*^ *: One of the students has said to me “Because it’s very pressured, especially on a Friday, to get back from Friday prayer to lectures on time, can the lecture timetable be changed?”… I mean, because to change the whole timetable around one group of students’ prayer needs is quite tricky…How does one respond to that? It’s very tricky. We are a secular organisation.*

Prejudice by students towards other students was only mentioned in passing, with no specific examples given:

*IAdm* ^*m*^ *: Issues of faith certainly, we’ve seen an amount of bullying, harassment and bad behaviour around issues of faith, whether it’s a question of denomination, Shia and Sunni, things around that. Whether it’s about somebody’s perceived behaviour being in line with one’s faith or not; yes, we’ve seen an amount of that as well.*

Some felt staff may just be somewhat insensitive when it comes to students from different ethnicities to their own, rather than actively prejudiced against them:

*IAdm* ^*m*^ *: I don’t think there’s an active prejudice at work on people’s part; I think there’s a lot of insensitivity.*

Some potentially insensitive behaviour was reported as being from students to staff, with the perception of some students that Black male staff are ‘scary’ and not wanting to ask them questions:

*IAca* ^*f*^ *: Another aspect of discrimination I’ve actually seen is from students to members of staff…there are a couple of Black lecturers and they have consistently been described as ‘scary’ to me [by White and Asian students], and this is a word that I have not heard described for any other lecturer and it’s been from multiple students who have described these people as scary. They just say their personality, their demeanour, how they present, they’re scary, and they haven’t said anything much more than that... And then I say, well, this person is head of the class and they’re perfectly nice, and so on and so forth, but no, no, they don’t want to talk to them, they want to talk to someone else instead.*

Staff may also be reluctant to discuss ethnicity-related topics, with a possible reason being that some are uncertain how to approach the subject, potentially leading to issues not being confronted:

*IAca* ^*f*^ *: I think probably academic staff are quite fearful of this issue, that they don’t know how to talk about it, they don’t know what’s politically correct. They’re scared of being corrected about it, and so they’re even uncertain about what terminology to use when talking about Black and ethnic minority issues…I’ve actually had members of staff say, “I don’t know what to call them. Is it OK to call them Black?” and things like that.*

## Discussion

Multiple issues were raised by students and staff that could potentially contribute to the BAME attainment gap. Both staff and students noted the importance of academic support networks, and the way course information is transferred through the social networks of students, which are often ethnically defined. This means those with larger social networks are at an advantage when compared with social minorities. Whilst previous research has found that attainment is linked to friendship groups, with ethnicity influencing the formation of these [19], a novel finding of this research is how important social networks are in the transfer of academic knowledge in medical school with course and exam resources transferred predominantly within these networks. Research by the National Union of Students (2011) found that Black students were more likely to feel like ‘the odd one out’ and be isolated and uncomfortable in their HE environment than other students, and if this is the case, then they are likely to be members of smaller social networks. This may have a particularly important impact in relation to resources such as academic notes and past exam papers. The University does not provide exam papers for all courses as the questions are often reused. Thus, those with fewer social links to more senior years are potentially biased-against. In such scenarios, educational institutions may benefit by having a centralised or regulated transfer of such academic information in order to create a more level ‘playing field’.

Another potential contributor to the attainment gap is that of behavioural change by BAME students, particularly those of Black ethnicity. Often, this was to counteract negative stereotypes they perceive as likely to be held by students and staff. Black students mentioned trying to overcompensate both academically and physically, saying that because they are Black, they are likely to be seen to be less academically proficient and less well-dressed than their White peers and thus they must prove this is not the case. These findings echo previous research, which revealed how some Asian medical students similarly felt they were being negatively stereotyped by staff, and as such were actively trying to combat this, for example by working extra hard [13]. The negative impact of these types of compensatory behaviours on academic performance has been explained by the theory of ‘stereotype threat’. This theory posits that perceived negative stereotypes reduce student confidence and result in increased anxiety, especially during examinations, which in turn hampers academic performance [13]. Therefore, actual stereotyping does not need to occur; the student need only think they will conform to a negative stereotype for it to have a detrimental effect on their performance. Nevertheless, it is clear that some students and staff are negatively stereotyping BAME students, by assuming certain behaviours due to their own preconceived ideas about these groups.

Some Black students felt they were being discriminated-against in examinations, particularly those that involve observation by examiners such as during OSCEs. A staff member also noted how some Black students sometimes modify their language in examination settings to unnecessarily try and speak how they think a doctor ‘should’ speak, which resulted in communication issues. Whilst previous research found no evidence of explicit examiner discrimination between BAME and White students in OSCEs, male BAME students who achieved poor marks were found to have different communication styles to their White counterparts, and it is suggested the examiners’ assumptions about what makes for good communication may have disadvantaged them [20]. A member of staff highlighted that examiners may have a Eurocentric view of how empathy is expressed, biasing against certain ethnicities of students; Black students commented on how examiners assumed that because they were not obviously blushing, they didn’t care about the exam and thus were marked-down. We are not aware of previous research having found this as a potential contributor to the attainment gap.

Asian students mentioned family responsibilities more often than other ethnicities; indeed some commented that they felt their family responsibilities are not shared by other ethnicities and that such matters are not understood by the University, potentially leading to a feeling of being disadvantaged against other students. Staff also mentioned that BAME students appear to have increased family ties than other students, and whilst it was felt that the University could perhaps do more to cater for their increased burdens, it was considered equally important to prepare students for professional life where they have to deal with balancing work and family commitments. We are not aware of previous research into the BAME attainment gap as having revealed this potential contributor.

The lack of ethnic diversity in the staff population was mentioned by both students and staff, with Black staff noted as particularly uncommon, however no student raised the lack of role models at the University as an issue, whilst staff suspected it may be. It is possible that students are finding role models in the University regardless of ethnicity, or indeed that role models are unimportant. However, ethnically-similar role models have previously been found to be crucially important in aiding the attainment of ethnic minority adult learners [9] and in making students feel empowered [10].

Other qualitative research has found that BAME students, especially those of Asian ethnicity, are more likely to be motivated by future career and course reputation and more strongly influenced by family than White students, who are more likely to be motivated by personal development and actual interest in the subject [13, 21]. Some staff participants seemed to think the same, believing that Asian students were more likely to have been pressured into studying these courses than their non-Asian counterparts. However, the student responses did not support this idea, with a wide variety of reasons given by students from all ethnicities and courses, and no particular dominance of the family influence for BAME students when compared with White.

This study has several strengths. It included both academic and administrative staff, as well as male and female students from a wide range of years, ages and ethnicities, including peer-led focus groups of ethnically-homogenous participants. The topic guides for the semi-structured student focus groups and interviews and semi-structured staff interviews included a mix of open-ended and closed questions, allowing them to bring up topics of particular importance to themselves, whilst also covering core areas.. The analysis, which was performed by two researchers experienced in qualitative research, allowed both inductive and deductive approaches, and together, this approach increases the chances of capturing a diverse range of views.

There were also limitations. The gender, ethnicity and age of the interviewer (HC) may have impacted how both students and staff responded in the interviews, with some perhaps uncomfortable discussing some topics in greater depth than others. Whilst the impact is not quantifiable and no participant stated they did not wish to speak further on a subject due to HC’s characteristics, it is nevertheless a possibility. HC noted that during the course- and ethnically-homogenous student-led focus groups, the discussions were more open with more ‘blunt’ language used when speaking directly about ethnicity when compared with the interviews, which supports similar findings by others [13, 16]. However, as there were no mixed ethnicity focus groups held, our conclusions can only be tentative. Furthermore, the more casual nature of the discourse may have also been due to the participants being familiar with the focus group leads. Equally, the presence of acquaintances may have limited some participant’s willingness to discuss deeply personal or sensitive issues that otherwise may have been raised in a one-to-one interview. The lack of the remaining planned focus groups was unfortunate, but we do not think this biased the outcomes as we held multiple interviews with students from a wide range of ethnic groups studying on the courses included, and as such think it unlikely that not holding the additional focus groups had any meaningful impact on the findings.

The academic attainment of student participants was not taken into account when recruiting participants for the study, meaning it is possible that participants were not representative of all attainment levels. Indeed, Woolf et al. (2008) found that high achieving students were more likely to attend their study’s focus group than their low achieving counterparts.

No noticeable differences were found between specific ethnicity subgroups, however this could be due to the sample size and less representation of some ethnic subgroups. Anecdotal evidence from staff and students suggests that Black males and those identifying as of Muslim faith face numerous issues that may be unique to them; however, there are comparatively few Black male students at the University and selecting participants on the basis of religion was beyond the scope of this study.

We were unable to hold the three focus groups for Asian medical students, White biomedical sciences and medical students due to too few students being willing to participate. However, where possible those who wished to take part in the focus groups were instead offered a one-to-one interview, which may have allowed deeper exploration of individuals’ experiences than otherwise would have been possible in the focus group setting.

## Conclusions

This study contributes to the limited body of peer-reviewed, qualitative research into this important area, whilst both supporting and contrasting with previous findings. It is clear that some staff think parents of Asian students are more likely to have a negative impact on student course choice than for other ethnicities, however the student data do not support this. Evidence to support the potential for stereotype threat to be having an adverse impact on Black students was forthcoming from both students and staff, and this research provides clear examples of behavioural changes as a direct result of trying to combat stereotypes. Whilst previous studies have not found evidence of discrimination impacting upon attainment, this study has found that forms of discrimination, whether conscious or unconscious, may be negatively impacting the attainment of BAME students in examinations. The research also highlights the importance of social networks for the transfer of academic knowledge and the impact ethnicity may have on the formation of such networks, with issues around segregation and the sharing of information outside defined groups. Students with increased family responsibilities, or who commute due to living with their families, perhaps because of parental control, financial issues or childcare, may be at a disadvantage to students living locally as they are unable to attend social and academic functions. The impact commuting has on BAME students in particular is therefore worthy of research in its own right.

Clearly there is a danger in referring to the BAME attainment gap as if it refers to a homogenous group – such a category is made up of multiple groups and is not a single entity with all individuals within having the same shared experiences, history and issues and the use of this term may be a limitation of all studies in this field at this time. Although, in this study we have identified some evidence of differences between specific ethnic groups here and this has to be borne in mind in any attempts to address this attainment gap. It is also difficult to know how many of the issues highlighted are specific to the University or are more widely applicable. There are implications for these findings in terms of addressing the attainment gap. For example, in terms of the content of training in cultural awareness for staff, the need for strategies to address ethnic segregation among students and unconscious bias training to address the issues identified here. Also, it may be important to make students aware of the actions that have been taken to try and counter biases in order to reduce the students’ perception of stereotyping.

## Additional files

Additional file 1: Student and Staff Interview Topic Guides. (DOCX 21 kb)

Additional file 2: COREQ Checklist. (DOCX 18 kb)

## Abbreviations

BAME: Black, Asian and Minority Ethnic
COREQ: Consolidated Criteria for Reporting Qualitative Research
HC: Hugh Claridge
HE: Higher education
ISOC: Islamic Society
KS: Khadija Stone
MU: Michael Ussher
OSCE: Objective Structured Clinical Examinations
SL: Sarah Lasoye
UK: United Kingdom
