# Supplementary material for: The ethnicity attainment gap among medical and biomedical science students: a qualitative study
Source: BMC Med Educ. 2018 Dec 29;18:325. doi: 10.1186/s12909-018-1426-5 (PMC6310969; doi:10.1186/s12909-018-1426-5)
Supplement: Supplementary file 1 — Student and Staff Interview Topic Guides. (DOCX 21 kb) [file 12909_2018_1426_MOESM1_ESM.docx]

# BME attainment gap topic guide

## Student Focus Groups and Interviews

### Transition to University

1. Who or what were the most influential factors in choosing the course you're studying?
2. How did you find integrating at the University?
   - Were there any barriers to integrating?

### Learning, teaching and assessment

1. What parts of your course do you most enjoy?
2. What have been the hardest/most challenging aspects of the course so far?
3. Who or what have been the most influential factors in your time at university thus far?
4. Do you feel comfortable interacting with lecturers during lectures, or do you speak to them privately, or avoid personal contact with them?
5. Do you feel comfortable with your lecturers’/staff members’ use of language, i.e. is it inclusive?
6. Do you feel the design of your course is representative of you? What changes would you like to see made, if any?
7. Do you feel comfortable taking part in seminars/CBLs/SBLs or similar group activities?
8. Do you feel your opinions are valued?
9. How would you describe your relationship with your personal tutor?
10. Can you describe your typical approach to studying?
    - What do you think are the most effective approaches to learning?
11. What do you think are the key factors that affect academic success at the University?
12. Have you experienced any barriers to your learning experience at the University?
13. Can you describe any teaching behaviour or styles that you feel have inhibited your learning?
14. Do you feel your appearance has impacted upon your learning experience here?
15. How do you feel about how you are evaluated? What changes would you like to see made?
16. Do you spend most of your time at the University/halls, or do you split your time back with your family
    - Do you have any outside-of-University work or family responsibilities?
17. Are there any cultural or identity differences you've felt at your time at the University, and have these impacted your learning experience?
    - Have you experienced discrimination or racism, directly or indirectly?

### Culture and student support

1. Do you feel the University is a welcoming and inclusive environment?
2. Have you ever felt you’ve been stereotyped whilst here at the University by staff, other students, your peers etc.?
3. Do you feel that you have had sufficient access to support services, such as learning skills assistance, counselling, career planning etc.?

## Staff Interviews

1. Are there particular groups of students you feel are better or worse prepared for university life than others?
2. Do you feel the University is a diverse, welcoming and inclusive environment for students? Please explain whether yes or no.
3. Is there an ethnic group you associate most with the lowest attainment?
4. Would you consider the ethnicity of a student as influencing how they perform in situations such as CBLs, SBLs, OSCEs etc.?
5. Are there ethnic differences in learning practice? What are they? How significant are they?
6. Do you feel you engage with all students similarly?
7. Can you identify any biases or prejudices that might affect your teaching, either consciously or subconsciously?
8. Can you describe the typical student who comes to you for additional help or support?
   - What sort of issues have they come to you with?
   - What do you think has caused them?
9. Have you ever been aware of discrimination against a student or group of students, either involving a fellow member of staff or student?
10. Do you feel you have had appropriate training to challenge racist or derogatory remarks in the University setting?
